# Determining confidence and anxiety of Australian community podiatrists in managing foot ulceration: A cross‐sectional study

**DOI:** 10.1002/jfa2.12037

**Published:** 2024-07-25

**Authors:** Naomi Anning, Jessica Stokes‐Parish, Helen Banwell, Ryan Causby, Annie Walsh, Peta Tehan

**Affiliations:** ^1^ School of Medicine and Public Health University of Newcastle Callaghan New South Wales Australia; ^2^ Faculty of Health Science and Medicine Bond University Gold Coast Queensland Australia; ^3^ Allied Health and Human Performance University of South Australia Adelaide South Australia Australia; ^4^ School of Clinical Sciences Faculty of Medicine Nursing and Health Sciences Monash University Clayton Victoria Australia; ^5^ College of Health Medicine and Wellbeing University of Newcastle Ourimbah Ourimbah New South Wales Australia

**Keywords:** anxiety, confidence, diabetic foot, foot ulcer, podiatry, survey

## Abstract

**Background:**

Diabetes related foot ulcer (DFU) is a leading cause of impaired quality of life, disability, hospitalisation, amputation and mortality in people with diabetes. It is therefore critical that podiatrists across all settings, including community settings, are confident and capable of providing care for diabetes‐related foot complications. This study aims to describe current practice, confidence and anxiety levels of community podiatrists in the management of patients with foot ulceration. Furthermore, current barriers to service provision and interest in future educational opportunities will also be explored.

**Methods:**

An online cross‐sectional survey was distributed to Australian community podiatrists. Descriptive variables including gender, age, professional experience, practice location and practise setting were elicited. A modified competitive State Anxiety Inventory‐2 (CSAI‐2) was utilised to measure anxiety related to managing a foot ulcer. Other questions included a combination of multiple choice and open‐ended free‐text responses relating to assessment, confidence and referral pathways.

**Results:**

One hundred and twenty‐two Australian community‐based podiatrists responded to the survey. A variety of ulcer sizes and complexity were reported to be managed in community settings. Confidence in DFU management was high in most manual skill domains including: stabilisation of the foot (85.7%, standard deviation [SD] 17.42), scalpel control (83.0%, SD 20.02), debridement with a scalpel (82.7%, SD 18.19) and aseptic technique (81.0%, SD 18.62, maintaining integrity of healthy tissue (77.3%, SD 21.11), removal of appropriate tissue (75.6%, SD 22.53), depth of ulceration (73.7%, SD 23.99) and ability to manage messy wounds (69.1%, SD 26.04). Curette debridement had substantially lower levels of reported confidence (41.0%, SD 34.24). Performance anxiety was low with somatic and cognitive anxiety of 6/24 and 3/8 on the CSAI‐2, respectively.

**Conclusion:**

Community podiatrists are managing foot ulcers of varying size and complexity. Confidence and anxiety do not pose a barrier to care. Adherence to wound assessment clinical guidelines is low and confidence with the use of curette was sub‐optimal. Further educational programs may overcome these barriers and support shared models of care between community and acute podiatry services.

**Abbreviations**
DFD: diabetes‐related foot disease
DFU: diabetes related foot ulcer
HRFC: high risk foot clinic
MDT: multi‐disciplinary team
RA: regional area

## 1 INTRODUCTION

Diabetes is one of the leading global burdens of disease affecting approximately 10.5% of the adult population or 536.6 million people worldwide [1]. Subsequently, diabetes related foot ulcer (DFU) is increasingly prevalent, with approximately 18.6 million people affected by DFU each year [2]. Diabetes‐related foot ulceration is a leading cause of impaired quality of life (QOL), disability, hospitalisation, amputation and mortality in people with diabetes [3, 4] with approximately 20% of individuals with DFU experiencing a lower extremity amputation [5]. In 2022, approximately 50,000 Australians had an active DFU, with a further 300,000 people considered at‐risk [4, 6, 7, 8]. Annually, $AU1.6 billion in health care expenditure is attributable to DFU with approximately 28,000 hospital admissions, 4500 amputations and 1700 deaths [8, 9, 10, 11].

Podiatrists have the expertise and skillset to positively impact outcomes related to diabetes‐related foot complications [12]. Podiatrists provide care that can improve QOL and enhance long‐term outcomes with a reduced rate of amputations and the overall healthcare burden [13, 14, 15]. Both local Australian and International guidelines highlight the importance of podiatry, inclusive of prevention through assessment and education, as well as acute management of foot complications = through delivery of optimal wound care, pressure offloading and conservative sharp debridement [16, 17]. In Australia, public sector or hospital‐based podiatrists are often well supported with access to resources and multidisciplinary teams or high‐risk foot clinics (HRFC) which enable holistic care to manage acute and complex limb‐threatening presentations. This leads to development of an advanced scope of practice. However, most of the Australian podiatry workforce work in community settings (65%), with the remaining 35% employed by the public health service [18].

Given that the podiatry workforce is largely based in private (community) settings and there are growing numbers of patients with diabetes‐related foot disease (DFD), it is foreseeable that community practitioners will increasingly be involved in the management of DFD. Shared‐care or collaborative care models are used extensively in diabetes and other high needs healthcare areas, including mental health and midwifery [19]. The fundamental principle of these models is that standard of care is the same wherever the care is delivered, there is multi‐disciplinary teamwork and empowerment of both patient and practitioner. Community podiatrists have an important role to play, however they likely face additional challenges, as they may not have equitable access to medical support, specialist equipment or clinical triage pathways. Previous Australian studies have demonstrated that there are significant differences in the assessment and management of individuals with DFU between public and community podiatrists, particularly in relation to assessment and the use of grading systems [20]. It is likely that community podiatrists have less frequent clinical exposure to acute DFU and therefore there may be differing skills and confidence. A previous randomised controlled trial by Banwell and colleagues identified that conservative sharp debridement of wounds was a considerable source of anxiety for podiatry students and that the use of simulation was effective at reducing anxiety and improving confidence [21]. It is unknown if these concerns remain in the community podiatry clinical workforce.

To date there is limited research describing emotional attributes that impact upon podiatrists' ability to manage DFU specifically, within aged care, community, or private practice settings. It is unclear whether a lack of confidence or heightened anxiety presents barriers to the management of DFU in primary health settings. Therefore, the primary aim of this study was to describe current practice, confidence and anxiety levels of community podiatrists (private practice, aged care or community settings) in the management of patients with foot ulceration. Furthermore, current barriers to service provision and interest in future educational opportunities will also be explored.

## 2 METHODS

### 2.1 Study design

This cross‐sectional online survey of Australian community podiatrists was conducted between 2022 and 2023 using Qualtrics® software. Practising resgistered Australian podiatrists based in the community, aged care or any private facilities were eligible to participate.

The survey instrument contained 17 items. Initial questions related to demographic data included participants' gender, age, years of professional experience, postcode, clinical practice and work setting. Geographical location was then classified into rural or metropolitan areas using the Australian Statistical Geography Standard‐Remoteness Area (ASGS‐RA) classification based on practice postcode [22].

The survey consisted of both multiple choice and open‐ended free‐text responses. A modified Competitive State Anxiety Inventory‐2 (CSAI‐2) [23] was embedded within the survey to determine self‐confidence and anxiety levels of respondents when managing patients with foot ulceration. This validated tool, in its modified version, has been utilised previously in podiatry research [24]. Additional questions enabling multi‐responses were purpose‐built to provide insight into further training opportunities which would be considered helpful in building skills and confidence in managing DFU (e.g., immersion in public practice, online learning or simulation). A copy of the complete survey is available in Additional file 1. Once the survey was developed, it was piloted among a small group of clinicians, with feedback provided to ensure questions were clear and clinically applicable. The survey was subsequently revised prior to dissemination.

### 2.2 Survey dissemination

The anonymous survey was approved by the University of Newcastle human research ethics committee (H‐2021‐0180) and all participants provided informed consent before completing the survey. Convenience sampling of the survey was used, with the survey distributed at a series of workshops (between 2022 and 23) and via snowballing in professional networks using social media accounts associated with the authorship groups and their affiliated associations.

### 2.3 Statistical analysis

Data were collated using Microsoft Excel (Microsoft Corporation) and statistical analyses were performed using SPSS V28 (IBM Corp, Armonk). Descriptive statistics were used to display variable data. Open‐text responses were analysed using inductive content analysis, performed by one researcher (NA), who read all responses and generated categories to provide a description of the responses. A second author (PT) reviewed and discussed these categories with the first author, with disagreements resolved by consensus when necessary. Multiple linear regression analysis was used to assess the relationships between years of clinical experience and confidence in applying different skill tasks. A *p* value of <0.05 was considered significant.

A multiple regression was run to explore the relationship between years of experience and confidence in performing various tasks (Table 1). Preliminary analyses were conducted to ensure no violation of the assumptions of normality, linearity, multicollinearity or homoscedasticity. There was independence of residuals, as assessed by a Durbin–Watson statistic of 2.18. There was homoscedasticity, as assessed by visual inspection of a plot of studentised residuals versus unstandardised predicted values. Multicollinearity was assessed by tolerance values greater than 0.1. The R2 for the overall model was 45.7%.

**TABLE 1:** Multiple regression of years of experience and confidence of skills.

Variable	Unstandardised B	Standard error	95% CI		Standardised coefficient beta	*p*	*R* ^2^	Adj *R* ^2^
Confidence debriding FU with scalpel	−0.095	0.071	−0.235	0.045	−0.184	0.180	0.46	0.21
Confidence determining FU depth	0.160	0.112	−0.061	0.381	0.371	0.155		
Confidence in ability to remove appropriate tissue	−0.100	0.115	−0.329	0.129	−0.219	0.390		
Confidence in integrity of healthy tissue with scalpel	0.089	0.069	−0.049	0.226	0.184	0.204		
Confidence with control of scalpel during FU debridement	0.124	0.078	−0.029	0.278	0.246	0.111		
Confidence in positioning/stabilising foot during debridement	−0.078	0.069	−0.214	0.059	−0.132	0.260		
Confidence with aseptic technique during debridement	0.042	0.057	−0.071	0.154	0.079	0.464		
Confidence in managing messy wounds	0.044	0.071	−0.097	0.185	0.113	0.540		
Confidence debriding FU with curette	−0.092	0.030	−0.151	−0.034	−0.323	0.002		

Abbreviations: CI, confidence interval; FU, foot ulceration.

## 3 RESULTS

### 3.1 Participant characteristics

In total, 122 Australian community‐based podiatrists responded to the survey. Table 2 shows the participant characteristics including demographics, employment profile and work setting. Seventy‐five (61.5%) participants were females, with a mean age of 37 years and mean of 10 years (standard deviation [SD] = 9.8) professional experience. Most participants (*n* = 104, 85%) were right‐hand dominant. Podiatrists from all states in Australia participated in the survey with the majority (*n* = 57, 47.5%) based in NSW. The majority of all participants were based in metropolitan centres (*n* = 84, 69.6%) with 19.7% (*n* = 23) and 5.7% (*n* = 6) based in inner and outer regional centres, respectively [22]. Less than 2.5% (*n* = 3) of participants were based in remote or very remote locations. The majority of participants surveyed worked in private practice settings (*n* = 91, 74.5%), with four participants reporting working predominantly in aged care, two podiatrists working in academia and the remainder (*n* = 25, 20%) not stating their primary place of work.

**TABLE 2:** Participant characteristics.

Demographics (*n* = 122)		
Mean age in years (SD)	37.5 (10.42)	
Sex (*n*, %)	Male	47 (38.5%)
	Female	75 (61.5%)
Mean professional experience in years (SD)	10 (9.8)	
Handedness	Right	104 (85.2%)
	Left	10 (8.2%)
	Ambidextrous	8 (6.6%)
Location	New South Wales	58 (47.5%)
	Queensland	23 (18.9%)
	South Australia	17 (13.9%)
	Victoria	13 (10.7%)
	Western Australia	6 (4.8%)
	Australian Capital Territory	2 (1.6%)
	Tasmania	1 (0.8%)
	Not recorded	1 (0.8%)
Geographical remoteness	Metropolitan (RA1)	85 (69.6%)
	Inner regional (RA2)	24 (19.7%)
	Outer regional (RA3)	7 (5.7%)
	Remote (RA4)	2 (1.6%)
	Very remote (RA5)	1 (0.8%)
	Not recorded	3 (2.5%)
Practice‐type	Private practice	91 (74.5%)
	Aged care	4 (3.3%)
	Other	3 (2.5%)
	Not recorded	24 (19.7%)

*Note*: Remoteness.

Abbreviation: SD, standard deviation.

### 3.2 Current work practices

**FIGURE 1:**
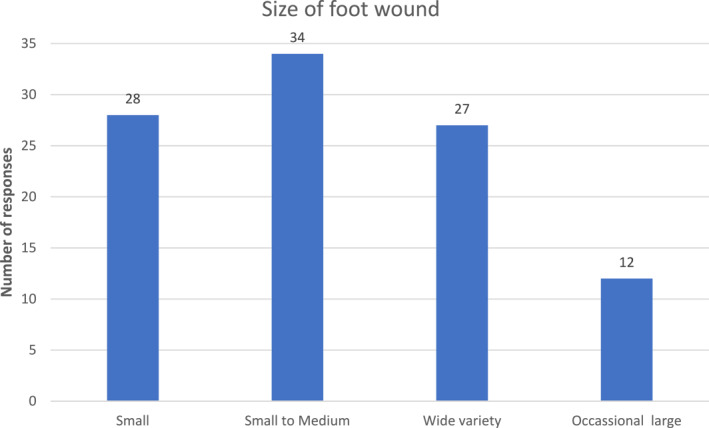
Size of wound in community practice.

All responding community podiatrists reported reviewing wounds, with most reporting seeing varying sizes (76.2%, *n* = 93) and varying severity (69.7%, *n* = 84). Small to medium wounds (28%, *n* = 34) were most prevalent with small (23%, *n* = 28) and a wide variety of sizes (22%, *n* = 27) also commonly reported. Only 12 (10%) participants indicated that occasional large wounds formed part of their practice (Figure 1).

**FIGURE 2:**
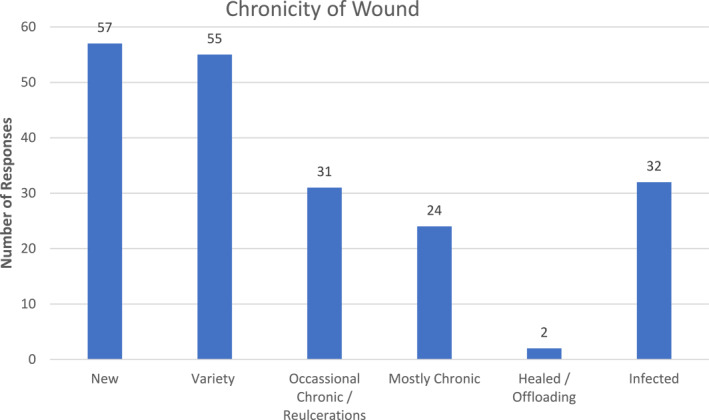
Chronicity of wound in community practice.

Figure 2 illustrates that community podiatrists most commonly deal with new wounds (47%, *n* = 57) and subsequently a variety (45%, *n* = 55) with infected (26%, *n* = 32) and chronic (25%, *n* = 31) wounds forming part of participant's practice.

**FIGURE 3:**
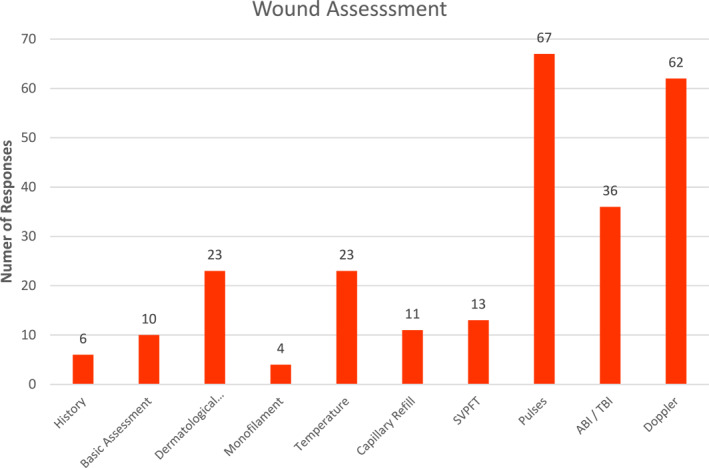
Assessment of wounds in community practice.

Participants were asked about what clinical assessments were performed as part of assessment of a patient with a foot ulcer. This demonstrated a wide variety of responses (Figure 3). In regards to vascular assessment methods, forty‐four (36%) podiatrists failed to report any form of vascular assessment. Pulse palpation was the most reported assessment response (55%, *n* = 67), followed by Doppler examination (54%, *n* = 62). Formal blood pressure measurements including ankle brachial or toe brachial indices were less frequently reported (29%, *n* = 36). A combination of three methods of vascular assessment were performed by 21 participants (17%), two methods by 44 participants (36%) and a single method by 10 participants (8%). Objective measures including both ankle brachial index/toe brachial index and Doppler were performed by 26 (21%) of the participants in this cohort. Low rates of physical assessments including capillary refill, temperature and Superficial Venous Plexus Filling Time were reported (Figure 3). Assessment for a loss of protective sensation with a 10 g monofilament was least commonly reported as part of a wound assessment, with four podiatrists (3%) reporting regular use as part of a wound assessment.

### 3.3 Inductive content analysis

Four key themes were derived from the descriptive data: infection; role & referral; collaboration; and barriers. A variable scope of practice was described with some podiatrists managing more complex wounds—both in size, severity and infection status; whereas others reported rarely being involved in wounds that were not acute. Additionally, the factors impacting on referral and engagement to public sector multidisciplinary teams was apparent, with many citing patient financial barriers as a key consideration driving onward referral, whilst others suggesting complexity was a key driver of referral. Sample quotes are outlined in Table 3. Podiatrists in this study reported debriding a median of 10 diabetes related foot ulcerations (IQR = 4–30) per annum. One hundred and twenty‐one podiatrists (99.2%) had performed sharp debridement of a foot ulcer using a scalpel, with a single participant reporting inexperience in scalpel debridement. Experience in curette debridement was less common with 34 (27.9%) podiatrists having previously utilised a curette in clinical practice.

**TABLE 3:** Theme table with supportive quotes.

Theme table with supportive quotes	
Infection	‘*Rarely manage infected wounds’*
	*‘Manage infected wounds occasionally’*
	*‘I refer infected wounds to HRFC health or GP if less severe’*
	*‘I have identified 5 cases of osteomyelitis in the last year’*
Role and referral	*‘Identify the issue, first treatment and stat's (TBI and doppler) and refer on’.*
	*‘Being the only podiatrist in town is the reason I get to see such a variety’*
	*‘There's also a big variety of GPs attitudes towards podiatrists and wound care’.*
	*‘I see new wounds and manage for 4 weeks in a rural setting, if no immediate reason such as probe to bone or ascending cellulitis and if no improvement they are referred to one of our MDT in the urban setting. Then share care with the MDT’*
	*‘Provide services to regional and rural areas where I would assess and treat a myriad of ulcers of different degrees of size, depth and colonisation. Bush mechanics!’*
	*‘Manage patients that are in remission following being in the public system. Naturally a number of these patients re‐ulcerate’.*
	*‘We handball between us and the public hospital for more serious larger or infected wounds’.*
	*‘Refer on the larger wounds, infected wounds or wounds in very high risk patients’*
	*‘Major wounds I request GP to refer off the HRFC’*
	*‘Manage them for a few weeks. If the wound shows no signs of improvement they are referred on to HRFC’*
	*‘I refer larger and more complex wounds onto HRFC’*
Collaboration	*‘Assist in chronic ongoing wound management in conjunction with GP or referred to our clinic for the community nurse’*
	*‘Chronic medium wounds in conjunction with HRFS’*
	*’Wounds are then referred on to the high‐risk podiatrist for review’.*
Barriers	*‘Chronic wounds of long‐time patients if they refuse to be referred onwards’.*
	*‘Refer to the HRFC (public system) if I do not feel that the case is within my scope of practice or there are financial barriers for the client to continue to come privately’*
	*‘Refer them to the local GP's for referral to the hospital. The reason is cost, in a private setting the patients are unwilling to pay for the dressings and appointments and therefore it is untenable’.*
	*‘Usually, I see them just until they can get into the local HRFC as it is too costly for most people to attend here regularly and we don't stock a huge range of dressings’*
	*‘Frequently see chronic, static wounds in which the patient has disengaged with the public system’.*
	*‘Vary to 10c size before sending to a HRFC/doctors due to the cost of redress in private practice’.*
	*‘I am the only podiatrist in XXXC so no HRFC to attend’*

Abbreviations: GP, general practitioner; MDT, multi‐disciplinary team; TBI, toe brachial index.

### 3.4 Confidence

**FIGURE 4:**
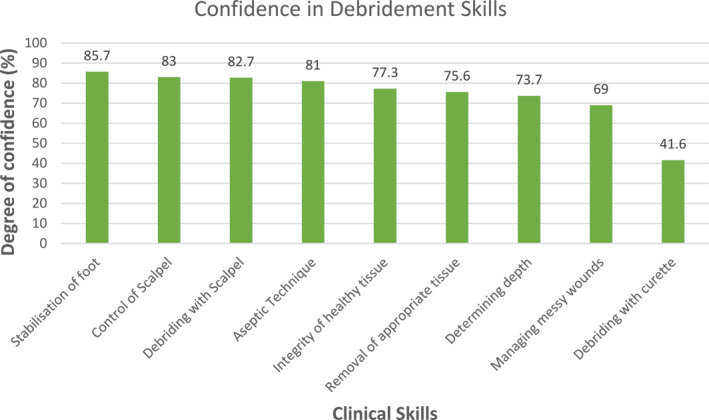
Confidence in debridement skills.

Overall, community podiatrists were confident in most clinical skills associated with DFU assessment and debridement, achieving >75% confidence in six out of the nine domains (Figure 4). Highest mean confidence was demonstrated in stabilisation of the foot (85.7%, SD 17.42), scalpel control (83.0%, SD 20.02), debridement with a scalpel (82.7%, SD 18.19) and aseptic technique (81.0%, SD 18.62). Marginally lower confidence was expressed for the ability to maintain integrity of healthy tissue (77.3%, SD 21.11) and removal of appropriate tissue (75.6%, SD 22.53) as well assessment of depth of ulceration (73.7%, SD 23.99) and ability to manage messy wound environments (69.1%, SD 26.04). Curette debridement had the lowest reported confidence with 41.0% (SD 34.24) overall.

Multi‐linear regression modelling demonstrated a significant positive relationship between years of experience and confidence levels (*F* = 9, 106) = 3.10, *p* = 0.002. Greater years of clinical experience was associated with higher confidence with use of curette and made a statistically significant contribution to the model (*p* = 0.002).

### 3.5 Anxiety

**FIGURE 5:**
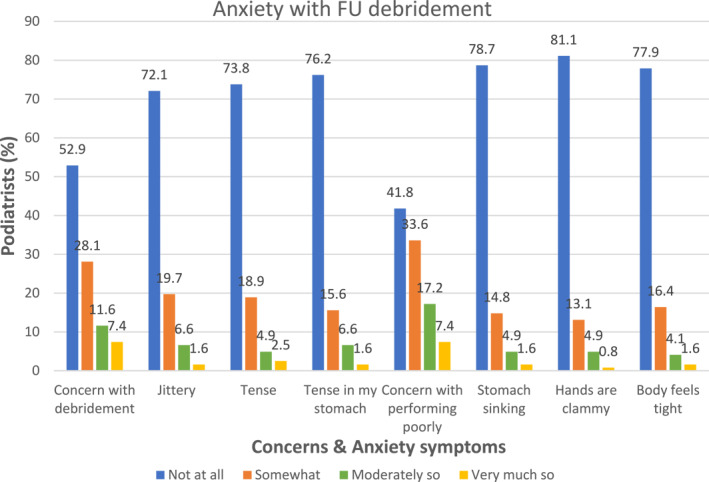
Anxiety with respect to FU debridement. FU, foot ulceration.

Performance anxiety was low with a median somatic anxiety overall of 6 out of 24 on the CSAI‐2. Cognitive anxiety was similarly very low with a median overall score of 3 out 8. Figure 5 demonstrates that the majority of participants reported low anxiety across all variables, with more podiatrists reporting moderate or greater levels of anxiety for performing debridement and concern with performing poorly.

### 3.6 Training opportunities

Most participants indicated an interest in future training opportunities with 86 (70%) participants indicating this is of interest. Online training videos were most sought after (*n* = 52, 43%). Attending day placement within a HRFC was also frequently reported (*n* = 49, 40%) and subsequent involvement in simulation training (*n* = 42, 34%).

Descriptive responses are illustrated in the following sample quotes:

Special courses in wound management

Knowing when and where to refer

Working in close partnership with public sector and knowing where our scopes overlap

One participant indicated that whilst they felt capable of managing wounds, providing patients with ‘*counselling or education is the main issue*’.

## 4 DISCUSSION

Podiatry has an essential role in preventing lower limb amputation in DFU [25]. Podiatry services are delivered in both community (primary health) and hospital (tertiary) settings, yet there is an apparent disconnect between the two services. Given the growing burden of disease and increasing pressures on hospital systems, shared models of podiatry care are increasingly being considered. It should first be understood if community podiatrists have the skills and ability to participate in shared care and what their needs are to confidently contribute to DFD patient care. The current study identified that podiatrists in primary settings are managing foot ulceration of varying size and complexity and generally have high levels of confidence and low levels of anxiety in managing foot ulceration. Furthermore, they conduct assessments in patients with foot ulceration; however, are not completely consistent with clinical guidelines. The most common reported barriers to managing foot ulceration in the community were the financial implications for the patient and the increasing complexity of the clinical case requiring medical or specialist input. Finally, community podiatrists described their educational needs in managing foot ulceration, outlining online courses and clinical placement (immersion) as preferable options for further upskilling in this area of practice.

### 4.1 Current practice and guidelines

The results of the current study reinforce that community podiatrists manage a variety of foot ulceration, with varying wound sizes, chronicity and complexity. Particularly apparent was that the majority of wounds managed in community settings were new wounds. This reinforces the role of community podiatrists as first responders in the DFU care pathway. It is therefore particularly important that this workforce is both confident and adequately skilled to manage this growing population. There was variability in the reported use of clinical assessments and subsequent referral patterns, with particularly limited general wound assessment and neurovascular assessment. A previous study by Quinton and colleagues also highlighted that community practitioners less frequently graded foot ulcer severity using guideline‐based recommendations [20]. The limited assessment reported in the current study is also consistent with a previous survey by Tehan and colleagues, which showed the majority of Australian podiatrists limited their vascular assessment to pulse palpation, rather than utilising ankle brachial or toe brachial pressure indices [26]. A lack of consistency and adherence to the Australian and international guidelines is likely to be underpinned by resource, time and/or financial constraints. Despite the variation in assessment, content analysis in the current study demonstrated that referral patterns were in keeping with current guidelines for both infection and wound complexity unless local limitations prohibited such referral. Standardisation of assessment and clinical pathways at local levels will likely improve consistency in triaging, escalation and referral, subsequently improving both patient experience and outcomes.

### 4.2 Barriers

The current study's findings identify that the greatest barrier to caring for individuals with DFU in the community was the perceived financial implications to complex ‘high cost, high needs’ patients with multiple comorbidities. Specifically, community podiatrists indicated that the clinical time required to manage the patients and the costs of consumables, including wound dressings, impacted on their ability to provide optimal care and for the patient to afford their care. Peak bodies including Diabetes Feet Australia and Wounds Australia have advocated for the Australian government to improve funding for DFD given that it is the second most common diabetic complication but ranks only 10th in terms of funding [9]. The recent announcement of the chronic wound consumables scheme by the Australian Commonwealth Government will remove one of the most significant barriers to providing wound care in primary health settings: the cost of wound consumables to the patient with a chronic wound [27]. Therefore, patients are increasingly likely to seek care for DFU from podiatrists in community settings.

The current study outlined that a lack of confidence or anxiety was not a substantial barrier to managing DFU by podiatrists in the community. Overall, self‐reported confidence levels in managing DFU are high. The clinical skill with lowest reported confidence was utilisation of a curette. However, greater years of experience was correlated with higher levels of confidence. This finding is likely related to a lack of exposure to curette use in wound care during entry‐level podiatry training, as current training tends to focus on scalpel skills [28]. Further training with curette could be achieved through the use of educational simulation and embedding of this manual skill within entry‐level curricula.

Geographical disparities in care provision were also highlighted by some respondents who classified themselves as *‘bush mechanics’*, highlighting the lack of available HRFC in their local area. This lack of specialised service subsequently forced the community podiatrist to take on the additional required care that the patient required, despite reporting not being adequately resourced to do so. Most HRFC are located in tertiary hospital settings in metropolitan areas of Australia, which leaves rural and regional podiatrists faced with attempting to provide specialist care in primary settings [29]. It has been established that individuals with foot ulceration living further from HRFS are more likely to undergo lower extremity amputation [30]. Therefore, more integrated models of care that support community podiatrists in rural areas to deliver appropriate care is urgently needed. Modern technology has a critical role to play in supporting community podiatrists and bridging the gap between community and public, urban and regional/rural service provision. Accessible telehealth and shared electronic records would promote a shared‐care model hypothetically enabling high‐quality integrated patient care. This is supported by previous research by Graham and colleagues, who also expressed an urgent need and called for investment in equipment, processes and training requirements essential to support practical use of telehealth in DFD management [31].

### 4.3 Educational opportunities

Participants in this study were enthusiastic to pursue further learning opportunities to enhance practical knowledge and skills in relation to foot ulcer management in the community. Online courses were the most popular preference (*n* = 52), perhaps driven by the dramatic shift towards online educational tools during COVID‐19. Federal funding initiatives towards flexible and accessible online educational opportunities support learning and upskilling in the current workforce are necessary. Furthermore, many respondents (*n* = 49, 40%) demonstrated a willingness to attend day placement (clinical immersion) within a public HRFC. One participant stated *‘working in close partnership with public sector and knowing where our scopes overlap’* would be of great interest. This would likely lead to improved professional clinical pathways and relationships within a shared‐care model. Finally, simulation could be a useful tool to further enhance skills and clinical reasoning in managing foot ulceration in community settings. A previous study by Lazzarini and colleagues demonstrated that simulation was effective at generating interest and subsequently enhancing clinicians' confidence, knowledge and satisfaction in managing DFU [32]. Future educational opportunities for community podiatrists should focus on guideline‐based assessment, triage and establishment of clinical pathways with the local HRFC.

### 4.4 Recommendations

The current study has elucidated some of the current gaps in the provision of DFD care in community settings in Australia. In order to progress with shared models of care for DFD patients, some key recommendations can be made based on these findings to better support community podiatrists. Firstly, greater educational support for community podiatrists to grow their clinical skills and knowledge in managing patients with DFU is needed. This education should focus on guideline‐based assessment, appropriate triage and escalation and building and maintaining clinical pathways to tertiary care. In order to provide equitable and convenient access to education across the country, these resources should be made available online where possible to ensure that rural practitioners also have access to these resources. Secondly, clinical placements or immersions could be offered to community podiatrists in HRFC to assist practitioners to build relationships with their local HRFC and further grow clinical skills by observing management of complex, acute foot disease. Finally, the use of a curette, a simple but effective clinical tool should be embedded in podiatry entry‐level curricula to increase confidence of podiatrists using this tool in patients with DFU.

### 4.5 Limitations

One key limitation of this study is the small number of survey participants, representing approximately 3% of the 3600 registered podiatrists in community settings practising throughout Australia [33]. Therefore, whilst the data accurately portrays the participants, these results may not be generalisable to the entire Australian podiatry workforce. However, the high female proportion (61%), mean age (37.5 years) and professional experience (10 years) are reflective of the Australian‐registered podiatry workforce[33]. Future studies could investigate different recruitment methods, such as social media platforms such as Instagram® or TikTok ® to improve engagement and response rates. Furthermore, 24 participants in this study did not specify the nature of the practice in which they currently worked with it hypothesised that some podiatrists in this study worked across more than one type of practice. Secondly, the sampling technique used may have caused sampling bias. Whilst the survey was snowballed and advertised online, it was also disseminated at education events attended by podiatrists. Therefore, it could be suggested that participants from these educational events may have had greater interest in diabetic foot management and continuing education activities. It could be purported that the survey was self‐selected with less confident podiatrists choosing not to participate. Whilst the survey contained a validated tool to measure confidence and anxiety, the complete survey was not validated. This should be taken into consideration when interpreting the results of this study. Finally, as the study was primarily aimed at assessing confidence and anxiety, podiatrists may have been less accurate in reporting standard assessment techniques, resulting in under‐reporting of some outcomes pertaining to assessment. Future studies could consider taking a more comprehensive approach to describing current practice.

## 5 CONCLUSION

This study identified that community podiatrists are frequently managing foot ulceration in primary settings of varying severity and complexity. They are confident in their clinical skills but are not consistently applying assessment in‐line with national and international guidelines. Common barriers to providing care of patients with foot ulceration were the financial capacity of patients to afford the clinical consult time and consumables required for wound care. There is an opportunity to provide structured education including clinical immersion to support community podiatrists to gain skills in assessment, triage and establish care pathways with their local HRFC. Podiatrists in regional and rural areas would particularly benefit from the use of telehealth to support their provision of evidence‐based clinical care in this population.

## AUTHOR CONTRIBUTIONS

**Peta Tehan**: Conceptualization, data curation, formal analysis, investigation, methodology, project administration, writing – review and editing. **Naomi Anning**: formal analysis, investigation, writing – original draft. **Helen Banwell**: Data curation, methodology, writing – review & editing. **Ryan Causby**: Methodology, writing – review & editing. **Jessica Stokes Parish**: Conceptualization, methodology, software, writing – review & editing. **Annie Walsh**: Data curation, investigation, writing – review & editing.

## CONFLICT OF INTEREST STATEMENT

The authors declare there are no conflicts or competing interests.

## ETHICS STATEMENT

This research was completed under the ethical approval of the University of Newcastle ethics committee approval number H‐2021‐0180.

## CONSENT FOR PUBLICATION

Not applicable.

## Data Availability

De‐identified data is held securely with the lead author and may be available upon request.
